# Ixodid ticks of traditionally managed cattle in central Nigeria: where *Rhipicephalus* (*Boophilus*) *microplus* does not dare (yet?)

**DOI:** 10.1186/1756-3305-6-171

**Published:** 2013-06-07

**Authors:** Vincenzo Lorusso, Kim Picozzi, Barend MC de Bronsvoort, Ayodele Majekodunmi, Charles Dongkum, Gyang Balak, Augustine Igweh, Susan C Welburn

**Affiliations:** 1 Division of Pathway Medicine, Edinburgh University Medical School, The Chancellor’s Building, 49 Little France Crescent, Edinburgh, EH16 4SB, UK; 2 The Roslin Institute and Royal (Dick) School of Veterinary Studies, Easter Bush, Midlothian, EH25 9RG, UK; 3 Nigerian Institute for Trypanosomiasis Research, PMB 03, Vom, Jos, Plateau State, Nigeria

**Keywords:** Cattle, Ticks, Tick-borne diseases, sub-Saharan Africa, Nigeria

## Abstract

**Background:**

Ticks and tick-borne diseases (TBDs) undermine cattle fitness and productivity in the whole of sub-Saharan Africa, including Nigeria. The aim of this study was to document the composition of tick species, assessing the burden of infestation, in traditionally managed cattle in an area of central Nigeria where acaricides have not been used historically.

**Methods:**

The study was carried out in September 2010 in 9 villages belonging to three neighbouring local government areas in Plateau State, Nigeria. In each village all visible adult ticks were collected from at least 15 cattle (mean number = 25). Collected ticks were preserved in 70% ethanol to be counted and morphologically identified to the species level.

**Results:**

A total of 5011 ixodid ticks (1935 males and 3076 females) were collected from 228 cattle, comprising 14 calves, 33 juveniles, and 181 adults. Three tick genera (i.e., *Amblyomma*, *Hyalomma*, and *Rhipicephalus*, including the *Boophilus* sub-genus) and 11 species were identified. The most prevalent species was *Rhipicephalus* (*Boophilus*) *decoloratus* (41.4%), followed by *Rhipicephalus* (*Boophilus*) *annulatus* (15.4%), *Rhipicephalus guilhoni* (12.0%), *Rhipicephalus* (*Boophilus*) *geigyi* (7.6%), *Hyalomma truncatum* (7.4%), *Amblyomma variegatum* (6.3%), *Rhipicephalus simus* Group (4.0%), *Rhipicephalus turanicus* (1.2%), *Rhipicephalus sanguineus* (0.3%), *Hyalomma rufipes* (0.2%), and *Rhipicephalus lunulatus* (n = 1). Mean tick loads recorded were relatively high (22 ± 1.4), in spite of the practice of hand removal of ticks traditionally undertaken by the Fulani pastoralists in the area. Calves bore a significantly lower tick burden than adults (p = 0.004). *Rhipicephalus* (*Boophilus*) *microplus* was not found in the area, suggesting that the eastbound expansion of this tick species in West Africa, has not yet reached central Nigeria.

**Conclusions:**

This study ascertained the presence of a broad variety of cattle tick species, most of which are of veterinary importance. The presence of each tick species is correlated with the potential occurrence of tick-borne pathogens and suggestions for tick control in the area are considered. Results should assist the diagnosis of related TBDs in cattle as well as the strategic planning of cost-effective tick control.

## Background

Ticks are ranked as the most economically important ectoparasites of livestock in the tropics, including sub-Saharan Africa (SSA) [1]. Their veterinary importance is related to their blood-feeding, from which both their direct and indirect pathogenicity originates [2]. In cattle, tick infestation alone can cause anaemia, stress, reduction in weight gain and milk yields, depreciation of hide value, hypersensitivity and toxicosis, leading also to secondary infections [2]. In addition, some tick species can act as vectors of pathogens causing a number of tick-borne diseases (TBDs), a serious impairment to cattle health and productivity in SSA [3].

In Nigeria, 90% of the cattle population is kept under the traditional pastoral husbandry of Fulani herders; mostly concentrated in the central-northern part of the country [4]. Under the Fulanis’ management, cattle are extensively grazed in pastures and forest, and exposed to infestation by the three tick genera present in Nigeria (i.e., *Amblyomma*, *Hyalomma*, and *Rhipicephalus* spp., sub-genus *Boophilus* spp. included) [4-7]; genera are known vectors of the causative agents of the most important bovine TBDs in West Africa: anaplasmosis, babesiosis, ehrlichiosis (cowdriosis) [8]. Usually low in the dry season, tick loads on cattle tend to increase after the first scattered rains, reaching the highest abundance one month after the heavy rains (i.e., from July to September), when all tick species are expected to be present [7,9-11]. The associated tick-borne infections are endemic in the indigenous (*Bos indicus*) cattle population [8,12], and are responsible for chronic rather than acute disease symptoms. Nevertheless, TBDs may become clinically apparent in particular circumstances of malnutrition or debilitation by a concurrent disease (e.g., trypanosomiasis) [4,10], or during the wet season, in the presence of a high tick challenge [7]. Furthermore, TBDs also represent a major limitation to the improvement of cattle production given the high morbidity and mortality rates they can cause in more productive, but susceptible, exotic (*Bos taurus*) cattle breeds, when introduced for crossbreeding purposes [13].

Ticks on cattle are perceived as a hazard by the Fulani pastoralists, who traditionally control them by manual removal three times a week during the wet season (i.e., April to October) and twice a week during the dry season (i.e., November to March) [6,10]. Neither dip tanks nor acaricides have ever been used in this part of the country [10].

Knowledge of tick distribution is an essential pre-requisite for devising any effective control of these arthropods and the infections they transmit [14]. Existing information on tick infestation of cattle in Nigeria is rather out-dated [5,7,9,15,16], mostly derived from studies carried out in the south of the country [5,15]. The only work published to date on central Nigeria focused on the seasonal dynamics of *Amblyomma variegatum*, without identifying the other specimens retrieved any further than the genus level [7]. West African cattle are currently threatened by the expansion of the harmful and invasive tick species, *Rhipicephalus* (*Boophilus*) *microplus*, seemingly imported from Brazil and found so far only in the Ivory Coast and Benin [17]. Ascertaining the distribution of *Rh. (Bo.*) *microplus* in this area is important, as this species is the vector of the bovine pathogen *Babesia bovis*[18], and is also resistant to acaricides [19].

The aim of the present work was to document the tick species infesting cattle in central Nigeria, assessing the infestation rate of surveyed animals, at a time of the year (i.e., wet season) when the tick load on the host is known to be most abundant [7].

## Methods

### Study area

**Figure 1:**
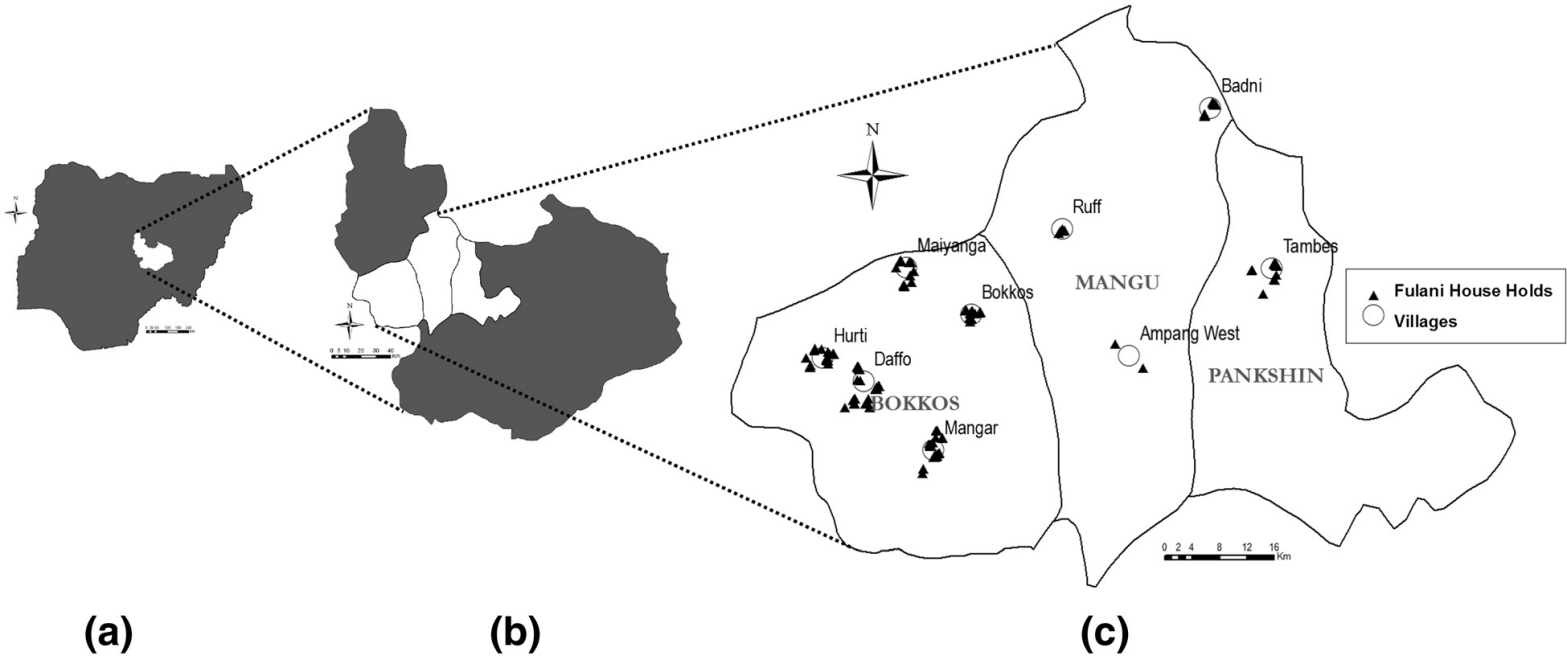
**Map of the study area.** (**a**): Federal Republic of Nigeria; (**b**): Plateau State; (**c**): local government areas with the 9 study villages.

The study was carried out in the second half of September 2010 in 9 villages belonging to three neighbouring local government areas (LGAs), namely Bokkos, Mangu, and Pankshin, in the central part of Plateau State, Nigeria (Figure 1). The study area covered 142 km^2^, ranging between latitude 9°14′ and 9°59′ N and longitude 8°79′ and 9°38′ E at an average altitude of 1280 m. All villages are in the sub-humid region of Nigeria, with the dry season generally extending from November to April, and the wet season from April-May to October. The rainfall pattern is mono-modal, with most (~80%) of the rains occurring between June and September. Annual rainfall is ~1400 mm and the daily mean temperature ranges between 18 and 22°C [20].

All cattle reared in the area are of autochthonous (*B. indicus*) genotype, with the vast majority (~80%) belonging to the White Fulani breed, and with a small number of either Bunaji or White Fulani x Bunaji crossbreeds. Cattle are grazed on communal pastures year-round according to the traditional Fulani herding system. Other livestock species raised in the area include sheep, goats, poultry, and dogs. No chemical acaricides are used under the Fulani farming system.

### Tick sampling and identification

In each village, all visible adult ticks were collected from at least 15 randomly selected cattle varying in age and sex, all belonging to the indigenous (*B. indicus*) White Fulani breed. Tick collection was performed using blunt steel forceps, by thorough examination of the entire body surface of the animals. Ticks from each animal were stored separately in vials containing 70% ethanol, labelled with information on the host (i.e., sample number, age), village, and date of sampling. Age of the animals was estimated on the basis of the dentition score method developed for zebu cattle under a low plane of nutrition [21] and on information provided by their owners. Once quantified, each animal’s age was recorded either as ‘calf’ (0–6 months), ‘juvenile’ (6–24 months), or ‘adult’ (older than 24 months). Once in the laboratory, all collected ticks were counted and identified to the genus and species level using a stereomicroscope (up to 100× magnification) and following the morphological keys in Walker *et al.*[22]. For those belonging to the genus *Rhipicephalus*, keys by Walker *et al*. [23] were also used.

### Statistical analysis

Statistical analysis was performed using R software (http://www.r-project.org). Prevalence of each tick species was calculated with the exact binomial 95% confidence interval using the reciprocal of the sample size, with the ‘survey’ package in R. Confidence intervals were calculated by the use of the svymean() function and adjusted with the svyciprop() function. Mean tick counts and their standard error (± SE) were calculated for each village, age group, and tick species. Cumulative counts were statistically compared according to age groups of cattle using the Kruskal-Wallis test. Post hoc analysis was then performed using the Holm P value adjustment method in a pairwise Wilcoxon rank sum test. P values <0.05 were considered statistically significant.

The study was carried out with the full approval of cattle keepers and the federal government body, the Nigerian Institute for Trypanosomiasis Research (NITR). The University of Edinburgh is a charitable body, registered in Scotland, with registration number SC005336.

## Results

A total of 228 cattle were checked for tick infestation in 9 villages (average = 25 animals/village). The population sampled consisted of 14 calves, 33 juveniles, and 181 adults. All animals were infested with adult ticks. A total of 5011 adult ixodid ticks (1935 males, 3076 females) were collected (Table 1). Mean tick counts recorded per village were relatively high (i.e., 22 ± 1.4), ranging from 7.6 (±1.5) in Ampang West to 46.5 (±7.91) in Mangar (Table 1). Three tick genera (i.e., *Amblyomma*, *Hyalomma*, *Rhipicephalus* including *Boophilus* sub-genus) and 11 species were identified. *Rhipicephalus* (*Boophilus*) *decoloratus* (Koch, 1844) was the most prevalent species (41.4%), followed by *Rhipicephalus* (*Boophilus*) *annulatus* (Say, 1821) (15.4%); *Rhipicephalus guilhoni* Morel & Vassiliades, 1963 (12.0%); *Rhipicephalus* (*Boophilus*) *geigyi* (Aeschliman & Morel, 1965) (7.6%); *Hyalomma truncatum* Koch, 1844 (7.4%); *Amblyomma variegatum* (Fabricius, 1794) (6.3%); *Rhipicephalus simus* Group Koch, 1844 (4.0%); *Rhipicephalus turanicus* Pomerantsev, 1936 (1.2%); *Rhipicephalus sanguineus* (Latreille, 1806) (0.3%); *Hyalomma rufipes* Koch, 1844 (0.2%). Only one male specimen of *Rhipicephalus lunulatus* Neumann, 1907 was retrieved. 4.1% of adult specimens were identified as *Rhipicephalus* (*Boophilus*) spp. but damaged mouthparts prevented identification any further than the sub-genus level (Table 2). *Rh.* (*Bo.*) *microplus* (Canestrini, 1888) was not found in the study area. All three boophilids, *H. truncatum*, and *A. variegatum* were retrieved in all nine villages. Male specimens outnumbered females for most species except for the boophilids and *Rh. sanguineus* (Table 2). A rather high individual variation was seen in terms of tick load, depending on the age and size of the animals. Calves were found to be significantly less infested than adults (p = 0.004), whereas no statistically significant difference was found comparing adults with juveniles (p = 0.2). Although not statistically significant (p = 0.2), the average proportion of ticks infesting juveniles was higher than the mean loads on calves (Table 3).

**Table 1:** Cumulative tick counts, mean tick loads ± standard error (SE) according to the villages sampled

**Village name**	**No. of cattle sampled**	**Total cattle population**	**No. of ticks collected**	**Mean tick count/animal ± SE**
Ruff	15	154	322	21.5 **±** 3.65
Mangar	15	1373	697	46.5 **±** 7.91
Tambes	16	854	301	18.8 **±** 4.4
Daffo	21	2933	686	32.7 **±** 5.6
Ampang West	22	790	168	7.6 **±** 1.5
Hurti	23	1011	594	25.8 **±** 4.3
Badni	27	383	851	31.5 **±** 3.3
Bokkos	36	2142	608	16.9 **±** 2.1
Maiyanga	53	2543	784	14.8 **±** 2.4
**Total**	**228**	**12183**	**5,011**	**22 ± 1.4**

**Table 2:** Cumulative counts, prevalence, number of males and females, and male: female ratio of ticks identified

**Tick species**	**Total**	**Mean prevalence%**	**Males**	**Females**	**Male: female ratio**
		**(95% confidence interval)**			
*Rhipicephalus* (*Boophilus*) *decoloratus*	1890	41.4	473	1417	1 : 3
		(36.5–46.3)			
*Rhipicephalus* (*Boophilus*) *annulatus*	819	15.4	189	630	1 : 3.3
		(11.9–19.0)			
*Rhipicephalus guilhoni*	434	12.0	302	132	2.3 : 1
		(7.2–16.8)			
*Rhipicephalus* (*Boophilus*) *geigyi*	306	7.6	45	261	1 : 5.8
		(6.1–9.1)			
*Hyalomma truncatum*	681	7.4	469	212	2.2 : 1
		(5.8–9.0)			
*Amblyomma variegatum*	361	6.3	245	116	2.1 : 1
		(4.5–8.1)			
*Rhipicephalus* (*Boophilus*) spp.	205	4.1	12	193	1 : 16
		(2.9–5.4)			
*Rhipicephalus simus* Group	239	4.0	155	84	1.8 : 1
		(2.5–5.5)			
*Rhipicephalus turanicus*	39	1.2	22	17	1.3 : 1
		(0.5–2.0)			
*Rhipicephalus sanguineus*	10	0.3	4	6	1 : 1.5
		(0.03–1.0)			
*Hyalomma rufipes*	26	0.2	18	8	2.2 : 1
		(0.1–0.4)			
*Rhipicephalus lunulatus*	1	<0.1	1	–	1 : 0
		(0.0–0.0)			

**Table 3:** Cumulative tick counts and mean tick loads ± SE of cattle according to age groups

**Age group**	**Cumulative counts**	**Mean tick load ± SE**
Calves (<6 months)	142	10.1 ± 2.7^a^
Juveniles (6–24 months)	595	18.0 ± 2.9
Adults (>24 months)	4274	23.6 ± 1.6^a^

^a^ Statistical significance between the two age groups.

The broadest diversity of tick species was encountered in adults, followed by juveniles, and calves. Boophilids, *H. truncatum*, and *A. variegatum* were more abundant in adults compared to juveniles and, more markedly, than calves (Table 4).

**Table 4:** Cumulative counts and mean loads ± SE of tick species according to age groups of cattle

**Tick species**	**Cumulative counts**			**Mean tick load ± SE**		
	**Calves**	**Juveniles**	**Adults**	**Calves**	**Juveniles**	**Adults**
*Rhipicephalus* (*Boophilus*) *decoloratus*	56	197	1637	4.0 ± 1.2	6.0 ± 1.7	9.0 ± 0.9
*Rhipicephalus* (*Boophilus*) *annulatus*	16	104	699	1.1 ± 0.5	3.1 ± 0.7	3.9 ± 0.4
*Rhipicephalus guilhoni*	27	54	353	1.9 ± 1.3	1.6 ± 0.7	1.9 ± 0.6
*Rhipicephalus* (*Boophilus*) *geigyi*	7	27	272	0.5 ± 0.2	0.8 ± 0.2	1.5 ± 0.2
*Hyalomma truncatum*	15	87	579	1.1 ± 0.4	2.6 ± 0.8	3.2 ± 0.4
*Amblyomma variegatum*	12	42	307	0.9 ± 0.4	1.3 ± 0.2	1.7 ± 0.2
*Rhipicephalus* (*Boophilus*) spp.	1	32	172	0.1 ± 0.1	1.0 ± 0.4	0.9 ± 0.1
*Rhipicephalus simus* Group	7	33	199	0.5 ± 0.4	1.0 ± 0.5	1.1 ± 0.2
*Rhipicephalus turanicus*	1	10	28	0.1 ± 0.1	0.3 ± 0.3	0.1 ± 0.1
*Rhipicephalus sanguineus*	0	7	3	0	0.2 ± 0.2	0.02 ± 0.01
*Hyalomma rufipes*	0	2	24	0	0.1 ± 0.04	0.1 ± 0.04
*Rhipicephalus lunulatus*	0	0	1	0	0	0.01 ± 0.01

## Discussion

The distribution of ticks within a specific habitat depends on several environmental and climatic factors such as annual rainfall, atmospheric temperature and relative humidity (RH), vegetation cover, altitude and host availability [24]. This study was carried out in the late wet season, when RH as well as the vegetation coverage, and therefore the abundance of adult ticks on cattle, are expected to be at their peak in central Nigeria [7,9,10]. This study aimed to assess the species diversity of ticks infesting cattle and their burdens; we only focused on the adult stages of these arthropods. Because of their small size, a large number of immature ticks can indeed be easily overlooked during field collection, resulting in a biased estimate of counts. Therefore, counts of adults can be taken as representative of the total infestation of all instars over the year, especially for three-host tick species, whose immature instars feed for short periods (e.g., four days) on cattle as well as on other hosts (e.g., small ruminants, wildlife, birds) [25]. In addition, larvae and nymphs of most genera lack the neatly distinctive morphological features needed for identification to the species level.

An average of 25 randomly selected cattle at each of the 9 villages were examined (Table 1). The greater number of adults rather than younger animals sampled reflects the age composition of Fulani herds, with at least 60% of cattle being adult [10]. The study ascertained the presence of a rather broad variety of tick species infesting cattle in central Nigeria, belonging to three genera (i.e., *Amblyomma*, *Hyalomma*, *Rhipicephalus* spp.) included in the Family Ixodidae. Five out the 11 species identified (i.e., *Rh.* (*Bo.*) *decoloratus*, *Rh.* (*Bo.*) *annulatus*, *Rh.* (*Bo.*) *geigyi*, *H. truncatum,* and *A. variegatum*) were retrieved in all study villages.

*Rh.* (*Bo.*) *decoloratus* was the most abundant species in the area, in accordance with previous work [5]. The Nigerian Jos Plateau seemingly provides an ideal environment for *Rh.* (*Bo.*) *decoloratus*, preferring highlands and sub-highlands receiving more than 800 mm of rainfall annually [26]. The second most prevalent species in this study was *Rh.* (*Bo.*) *annulatus*, previously found to be the most common tick attacking cattle in eastern Nigeria [15]. In Africa, the distribution of this tick is restricted to the northern and western part of the continent [22]. South of the Sahara, *Rh.* (*Bo.*) *annulatus* is associated with lowland rainforest and secondary grassland, with a clear increase in the vegetation cover after July-August [27]. Both *Rh.* (*Bo.*) *decoloratus* and *Rh.* (*Bo.*) *annulatus*, transmit *Babesia bigemina*[18], *Anaplasma marginale* and *Anaplasma centrale*[28], known to be endemic in Nigeria [8]. Being boophilids, one-host ticks that entirely develop on cattle after the egg hatch, their population is expected to be relatively constant throughout the year in this setting [9], presenting a constant threat of bovine anaplasmosis and babesiosis.

This study provides the first record of *Rh. guilhoni* in central Nigeria. Small numbers of adults of this tick were previously collected from the cattle during the rainy season in the far north of Nigeria [16]. Here, *Rh. guilhoni* was retrieved in 8 out of 9 villages and was the third most prevalent tick species (Table 2). As members of the *Rh. sanguineus* Group, this species is characterised by a more dense interstitial punctuation in the conscutum and a female genital aperture of a more truncated V-shape than the progenitor of its taxonomical group [23]. It is usually found infesting cattle, sheep, and camels, in steppe and savanna climatic regions [23]; its considerable presence on the Jos Plateau highlights the importance of assessing its role in pathogen transmission, as yet unknown.

This study also identified *Rh.* (*Bo.*) *geigyi* in central Nigeria. This species, only present in West Africa, is normally found in the savanna and forest zones of southern Nigeria, where it is the most abundant boophilid in the early dry season [5]. As this tick requires higher mean temperatures than *Rh.* (*B*o*.*) *decoloratus* and *Rh.* (*Bo.*) *annulatus*[27], it would be expected that the cooler conditions of the Plateau and, more in general, of the northern Guinea savanna woodland, would limit the expansion of its population into central-northern Nigeria. Although little studied in terms of pathogen transmission, *Rh.* (*Bo.*) *geigyi* could be of veterinary relevance in Nigeria, where it was proven to harbour piriform kinetes associated for shape and size with *B. bovis*, in both eggs and larvae that eventually infected splenectomised calves [29].

A number (n = 205) of boophilids were identified only as *Rhipicephalus* (*Bo.*) spp. due to partial rupture of their mouthparts, likely to have occurred at the time of collection, considering the small size and the short rostrum of these ticks. In particular, these were mostly engorged female specimens (see Table 2), whose feeding state did not allow the objective assessment of morphological features (e.g., shape of genital aperture), other than the mouthparts. The rostrum of boophilids bears species-specific features, such as the teeth rows in the hypostome and palp articles [22]. Nevertheless, as all these specimens had either one or both palps bearing a protuberance with or without an intact pectinate seta on article I, it was still possible to rule out the presence of *Rh.* (*Bo.*) *microplus* amongst them. The damage in their hypostome, though, did not allow the discrimination between *Rh.* (*Bo.*) *decoloratus* and *Rh.* (*Bo.*) *geigyi,* thus limiting the definitive identification to the sub-genus level.

The high number (n = 681) of *H. truncatum* recorded, reflects the seasonality of this tick in Nigeria, where it is known to peak in the late wet season [7,9]. The veterinary importance of this species is related to its ability to cause a toxic syndrome (sweating sickness), especially in young cattle [30].

**Figure 2:**
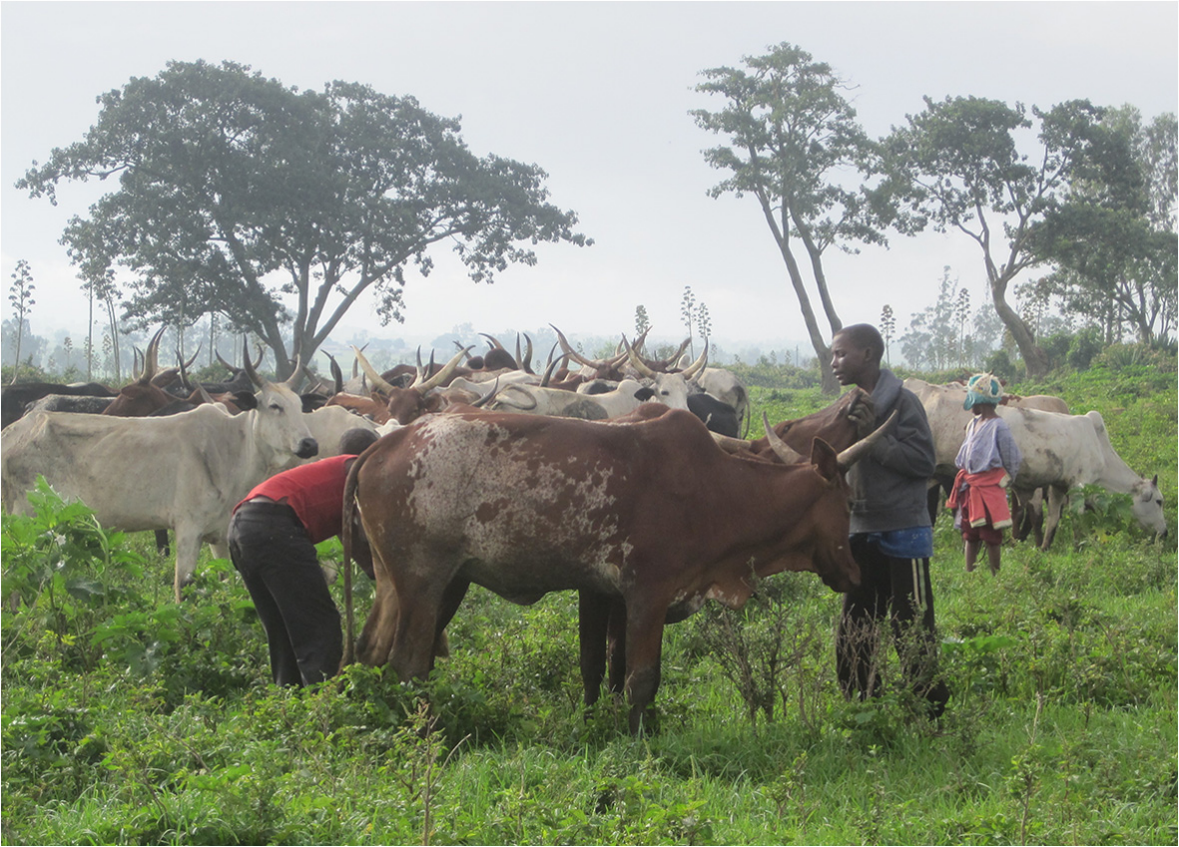
Young Fulani herders from the Plateau removing ticks manually from their cattle.

In a study carried out in the neighbouring state of Kaduna, central Nigeria, *A*. *variegatum* was the most prevalent species (>80% of all collected ticks) parasitizing cattle in September, followed by *Rhipicephalus* (*Bo.*) spp., and *Hyalomma* spp. [7]. The lower prevalence of *A*. *variegatum* (6.3%) recorded in the present study could be mainly attributed to the practice of hand-picking of ticks by the Fulanis, carried out up to three times a week during the wet season [6]. This control method mainly targets the most conspicuous *Amblyomma* adults, regarded as ‘koti’ (i.e., ‘dangerous ticks’ in Fulfulde language), by the local herdsmen, as opposed to the smaller *Rhipicephalus* and boophilid ticks that are consciously left attached, as they are believed to be ‘miri’ (i.e., ‘less harmful’) [7]. This operation is carried out when the animals are standing, when a number of body areas (e.g., groin, hooves, etc.) of the cattle cannot be easily reached; *H. truncatum* adults, that preferentially localize in the inter-digital clefts and the tail switch [31,32], are frequently overlooked (see Figure 2). In addition, although it keeps the animal to some extent free from ‘tick worry’, hand removal of ticks may not prevent transmission of tick-borne infections when not performed on a daily basis, as the transmission of pathogens may occur two days after the attachment of these arthropods to their hosts [33]. Due to their long mouthparts, *A. variegatum*, as well as *Hyalomma* ticks, can inflict serious cutaneous damage to cattle. Importantly, due to their preferential attachment to the udder and teats of cattle [31,34-37], infestation by both these tick genera may seriously hinder the suckling of calves. *A. variegatum* is of veterinary importance as it transmits *Ehrlichia* (*Cowdria*) *ruminantium*[38]*,* causative agent of heartwater and *Dermatophilus congolensis*, causing dermatophilosis [39], both known to be endemic in Nigeria. *A. variegatum* is also a vector of the mildly pathogenic, *Theileria mutans*[40,41] and *Theileria velifera*[42] both highly prevalent in Nigeria.

The paucity (n = 26) of adult *H. rufipes* collected in this study could indicate a small population of this species, known to be widely distributed in the most arid parts of tropical Africa [43]. Adults of *H. rufipes* are usually more numerous in the early part (i.e., June-July in Nigeria) than towards the end of the rainy season [44]. It is also possible that the altitude of the Jos Plateau might have acted as a further limiting factor to the establishment of an *H. rufipes* population*.* Considering that both larvae and nymphs of this two-host tick parasitize ground-feeding birds [45], it is likely that the adult specimens came from the moult of engorged nymphs brought to the study area by birds living in close contact with the herds (e.g., cattle egrets, oxpeckers, guinea fowls, etc.). Interestingly, no *H. rufipes* ticks were collected from calves (Table 4), suggesting that open pastures, grazed mainly by adult cattle, represent the most likely interface between cattle and these birds. Although scanty, the presence of this tick species is still of veterinary importance as it is known to transmit *A. marginale*[46], *Theileria annulata*[47], and *Babesia occultans*[48] to cattle. A relatively high number (n = 239) of adult *Rh. simus* Group ticks were collected from cattle of all age groups. Of the three taxa currently ascribed to the ‘*simus* Group’, only *Rhipicephalus muhsamae* Morel & Vassiliades (1965) was expected to be present in West Africa. However, in addition to the Group-specific punctuation pattern visible on the males’ conscutum, a number of morphological features (e.g., female genital aperture and shape of adanal plates) of the specimens collected in this study appeared closely related to the East African taxon *Rhipicephalus praetextatus* Gerstäcker, 1873. It was assumed these were the same specimens retrieved in the 1950s from several localities in central and northern Nigeria, identified as *Rhipicephalus simus simus*[49]. Usually found in regions with a savanna climate, the distribution of *Rh. simus* (or *Rh. simus simus*) is believed to be restricted to southern Africa [26], where the adults preferentially parasitize cattle, never reaching high loads [50]. As *Rh. simus* is a vector of *A. centrale*[51] and *A. marginale*[52] usually found in southern Africa, finding these specimens in Nigeria may be of epidemiological importance.

Other *Rhipicephalus* species collected include *Rh. sanguineus, Rh. turanicus*, and *Rh. lunulatus*. *Rh. sanguineus* in cattle has previously been recorded elsewhere [23,53] including Nigeria [15,54,55] and can be related to the presence of dogs, roaming freely within the boundaries of the villages where sampling took place, and in the vicinity of the cattle herds. This cosmopolitan three-host tick species is always associated with dogs, its preferential host, and the human-made dwellings where they live [56]. *Rh. turanicus* is usually more adapted to sheep and goats rather than cattle [57], and this might explain the small number (n = 39) of specimens collected in our study. Both *Rh. sanguineus* and *Rh. turanicus* are not known to transmit any pathogens to cattle [58]. Interestingly, only one male specimen of *Rh. lunulatus* was identified; this species has very distinctive morphological features (e.g., adanal plates’ shape in males; very broad U-shaped genital aperture in females) [59] compared to the other *Rhipicephalus* spp. ticks found in this survey. Adults of this three-host tick were previously reported in cattle in northern Nigeria, where they were found only during the first half of the wet season [16,49]. *Rh. lunulatus* is not regarded as a hazardous tick for cattle, although it was associated with a toxicosis causing paralysis in calves in Zimbabwe [60].

*Rh.* (*Bo.*) *microplus* was not found in the study area. We therefore assume that the eastbound expansion in West Africa of this invasive tick species, found first in 2007 in the Ivory Coast, and then in Benin [17], has not yet reached central Nigeria. This is of great epidemiological interest, as this tick species primarily parasitizing cattle, is known for being the competent vector of the highly pathogenic *B. bovis*[18]. Furthermore, the absence of *Rh.* (*Bo.*) *microplus* is also of interest in terms of tick control management, as this species is known to be highly resistant to several pyrethroid and organophospate compounds [19].

Males constituted the majority of specimens collected for most species (i.e., *Amblyomma, Hyalomma,* and *Rhipicephalus* spp.), with the exception of *Rh. sanguineus* and *Boophilus* spp. (Table 2). The male:female ratios recorded for most ticks coincide with data from previous work, with special reference to *Rh.* (*Bo.*) [25,61-64], *A. variegatum*[37,61,62,64], *H. rufipes*[62], but not for *Rh. sanguineus*[65]. With reference to *A. variegatum* the higher proportion of males rather than females collected is attributable to the biology of this tick species, known for localizing in preferential body areas (e.g., armpit, groin, udder, scrotum), forming typical clusters including a few females clasped by several males [31,32]. This is due to the release of aggregation-attachment pheromones (AAP) produced only by *A. variegatum* males, attracting unfed males and females [66] resulting in a concentration of more males than females on the attachment sites. The greater number of males than females collected for *Rhipicephalus* spp. is probably due to the fact that fully engorged female ticks are more easily groomed by the animals [67] and also drop to the ground earlier to lay eggs, while males tend to remain on the host for longer periods, feeding and mating several times before dropping-off [65]. This biological feature has been well documented for *Rh. sanguineus*[67], although in this study more females than males were collected, with a very low cumulative count (n = 10). The higher number of female rather than male boophilids collected is consistent with other studies [62] and likely reflects the relative difficulty in collecting the smaller males from hosts.

Here, the overall mean tick load recorded (i.e., 22 ± 1.4) was considered to be relatively high in the light of the hand-picking practice described above, which most likely reduced the actual number of adult ticks on the cattle sampled. It is also possible that the transhumance of weaned cattle according to the traditional Fulani herding might play a role in containing tick burdens as grazing areas are naturally spelled. Nevertheless, all the most hazardous tick species were recorded, although with different abundances (see Table 4), in all age groups in all study villages, with potentially large implications in terms of pathogen transmission.

In particular, this study revealed a pronounced effect of host age and size on the number of infesting adult ticks, especially when comparing calves (< 6 months) with adult cattle (>24 months of age) (Table 3). Although with no statistical significance, the mean tick loads of calves were also found at a lower proportion than those of juvenile cattle (6–24 months old), which bore lower burdens than the adults (Table 3). This finding is of interest considering that a significant amount of calving in the Fulani herds takes place in the early wet season (April-May) [10] and therefore, the calves sampled in this study have likely lived through the entire rainy season, in the presence of a high tick challenge. The significantly lower tick loads observed in calves as opposed to adults corroborates similar work carried out on indigenous cattle in SSA [68,69] including Nigeria [15,70]. The lower tick burdens recorded in calves could be indeed due to a combination of factors, including some form of innate immunity of indigenous cattle that decreases with age [71], the persistent grooming of calves by their respective dams [72], and the smaller body surface of younger animals compared to adults [73]. It could be argued, that animals with larger surface areas would possibly allow more contact opportunities for the ticks to attach themselves. This is also predicted by body size principle, according to which, the smaller the animal the fewer parasites (i.e., engorging ticks) it can afford to accumulate per unit of body surface because of the greater body surface to mass ratio [73]. Moreover, the lower tick burden recorded in young animals could also be due to the Fulanis’ practice of maintaining calves tethered together close to the homesteads, separated from the adult cattle. They therefore spend limited time grazing in the open grasslands with their dams, being possibly less exposed to the higher parasite burdens found on the pastures, driven by the higher host density.

## Conclusions

This study provides new information on tick populations in Nigeria and, more globally, in West Africa. The finding of *Rh.* (*Bo.*) *decoloratus*, *Rh.* (*Bo.*) *annulatus*, *A. variegatum*, and *H. truncatum* in all study villages is of great veterinary importance as these species are involved in the transmission of anaplasmosis, babesiosis by *B. bigemina* (*Boophilus* spp.); cowdriosis and dermatophilosis (*A. variegatum*) and sweating sickness (*H. truncatum*) [3]. Further studies are necessary to assess the occurrence of related TBDs in the Plateau State and would also help address the possible introduction of exotic breeds into the area.

*Rh.* (*Bo.*) *microplus* was not found in the present study, suggesting that this invasive and hazardous tick is not yet established in central Nigeria. Constant monitoring would, however, be advisable, as the Nigerian Jos Plateau provides favourable climatic and environmental conditions for the establishment of this tick species [27].

All animals sampled in this study were found infested with relatively high tick burdens. In order to be effectively implemented in the Jos Plateau, any strategic tick control should take into account the traditional farming system of the Fulani pastoralists. This could be achieved by combining the long-employed practice of manual removal of ticks with conventional control methods (i.e., acaricides) during the wet season when tick loads peak. In particular, in the light of inefficiency of the hand-picking method and that most of the calving takes place at the end of the dry season [10], the implementation of minimal or threshold tick control for adult female cattle, based on the application of spray or ‘pour-on’ acaricides to the udder region, might help prevent the topical attachment of *A. variegatum* and *Hyalomma* ticks, thereby improving milk yields [25]. Importantly, such a strategy would also preserve the endemic stability of the indigenous cattle herds in the area with regards to bovine TBDs [74]. Furthermore, while the indigenous White Fulani cattle are better able to bear these tick burdens, it is likely that exotic (*B. taurus*) or cross (*B. indicus* x *B. taurus*) breeds, if introduced in this area, unless subjected to intensive acaricide treatment, will become heavily infested with ticks and exposed to TBDs.

## Consent

The photograph was taken with the consent of the individuals portrayed and their families, as well as the community chief in the village of Maiyanga, Bokkos local government area.

## Competing interests

The authors declare they have no competing interests and the sponsors had no role in the study design, data collection and analysis, decision to publish, or preparation of the manuscript.

## Authors’ contributions

VL, KP, AM, AI, and SCW conceived of the study and participated in its design. AM coordinated the field activities. VL, GB, and CD carried out the tick collection. VL took care of tick identification. VL and BMCB carried out the statistical analysis. VL, KP, BMCB, and SW wrote the paper. All authors read and approved the final manuscript.
